# COMIT-ANZ: an audit of cholangiocarcinoma and mixed hepatocellular carcinoma-cholangiocarcinoma outcomes for patients undergoing liver transplantation in Australia and New Zealand

**DOI:** 10.3389/frtra.2026.1856922

**Published:** 2026-08-07

**Authors:** Georgina E. Riddiough, Kat Hall, Mandy Byrne, Kai Brown, Daniel Kilburn, Su Kah Goh, Matthew Marshall-Webb, Carlo Pulitano, Karen Chan, Adam Philipoff, Ali Nazaar, Eunice Lee

**Affiliations:** 1 Department of Surgery, The University of Melbourne, Austin Health Precinct, Heidelberg, VIC, Australia; 2 ANZLITR, Austin Health, Heidelberg, VIC, Australia; 3 Princess Alexandra Hospital, Woolloongabba, QLD, Australia; 4 Flinders Medical Centre, Bedford Park, SA, Australia; 5 Royal Prince Alfred Hospital, Camperdown, NSW, Australia; 6 Sir Charles Gairdner Hospital, WA, Australia; 7 Auckland City Hospital, Grafton, Auckland, New Zealand

**Keywords:** cholangiocarcinoma, hepatocellular carcinoma, liver neoplasms, liver transplantation, survival

## Abstract

**Introduction:**

Recent ILTS guidance supports liver transplantation for patients with very small intrahepatic cholangiocarcinoma. This guidance was informed mainly by North American and European data. The applicability of these guidelines to the region of Australia and New Zealand is unknown.

**Methods:**

A retrospective study was performed to measure clinical outcomes of liver transplantation in the ANZ region to ascertain the applicability of this new guidance to this region. Historic patients having undergone LT for known cholangiocarcinoma or incidentally detected cholangiocarcinoma or mixed type hepatocellular-cholangiocarcinomas on explant histology were identified via a national registry. Data was inputted from all transplant units within the ANZ region.

**Results:**

We found that 64 patients had either knowingly or unknowingly been transplanted for cholangiocarcinoma or mixed type hepatocellular-cholangiocarcinoma in our region.

**Discussion:**

Overall survival for patients with very small intrahepatic cholangiocarcinomas was 80%, in line with the current international data. We also report 5-year overall survival for patients with incidentally detected mixed tumours on explant histology was 68%.

## Introduction

Transplant oncology is a rapidly emerging subspecialty of clinical transplantation with the goal of improving clinical outcomes and survival for patients with solid organ malignancies that might otherwise be inoperable. Fundamentally, transplant oncology aims for complete resection of the cancerous organ *en bloc* followed by implantation of a healthy organ (1, 2). Renewed interest in this subspeciality of clinical transplantation raises important surgical and ethical issues regarding the utility of donor organs. Expanding the indication criteria for liver transplantation (LT) to include a variety of primary and secondary hepatic malignancies will increase demand for transplantation, which raises ethical and practical questions regarding how this demand can be met. Currently, there are established pathways for patients with a variety of primary and secondary hepatic malignancies including hepatocellular carcinoma, colorectal liver metastasis, cholangiocarcinoma, metastatic neuroendocrine tumours, hepatoblastoma and hepatic epithelioid haemangioendothelioma to undergo LT. Recent advances in machine perfusion (3, 4) and donation strategies such as normothermic regional perfusion (5–7) have facilitated expansion of the donor pool by improving clinical outcomes from marginal and donation after cardiac death donors. Additionally, expansion of living related donation programs may be another mode of increasing donor organ supply (8–11). In parallel, listing criteria for transplant oncology patients is also a key issue. It is imperative to consider how listing patients for LT for oncological indications impacts upon those non-oncological patients also waiting for LT. Work in this area is ongoing and patients with HCC currently receive exceptional MELD points to assist them in competing with non-oncological listed patients (12).

In particular, pathways for patients to undergo LT for cholangiocarcinoma (CCA) have evolved significantly in the last two decades. Cholangiocarcinoma is a tumour which originates from the bile duct epithelium and carries a very poor prognosis. These tumours are usually classified according to their site and can be intrahepatic (iCCA) or extrahepatic; the majority of extrahepatic CCAs are perihilar (pCCA). The site of the cholangiocarcinoma is an important factor in determining which pathway patients may access liver transplantation. Data supports LT in the setting of unresectable pCCA when transplant centres have an established neoadjuvant chemoradiotherapy protocol in place (13). 5-year overall survival of 58% has been reported for patients with *de novo* pCCA post LT (14). Additionally, several publications in the last two decades have supported LT as a treatment modality for very early iCCA (single tumour, <2 cm) in cirrhotic patients (15, 16) and this is reflected in the most recent International Liver Transplant Society (ILTS) guidelines (17).

Furthermore, mixed type hepatocellular-cholangiocarcinomas (HCC-CCAs) are rare tumours with histological features of both HCC and CCA that may be detected incidentally on liver explant histology post transplantation. These tumours occur at such low frequencies that at present, the clinical outcomes of LT for incidentally detected mixed HCC-CCAs is unknown. Pre-transplant diagnosis of mixed HCC-CCAs is challenging for multiple reasons. Radiologically, mixed HCC-CCAs cannot be clearly distinguished from pure HCCs and no pathognomonic radiological features have been identified (18). Biopsies and tumours markers also lack sensitivity for mixed type tumours. Elevated alpha fetoprotein and Ca19.9 may both occur and reflect the biphenotypic nature of these neoplasms (19). Publications addressing LT for mixed HCC-CCAs are scarce, however, studies have demonstrated that mixed HCC-CCAs are more aggressive than pure HCCs and associated with higher recurrence rates following liver resection (20, 21). Presently, no study has clearly shown a survival benefit for patients with HCC-CCAs undergoing LT and subsequently (22), advanced mixed HCC-CCAs are not a recognized indication for LT (17).

The majority of the data informing the current ILTS guidelines is derived from North American, Asian and European centres (17). This study set out to assess the clinical outcomes of patients undergoing LT for CCA or incidentally detected mixed HCC-CCAs in Australia and New Zealand and to assess the applicability of the ILTS guidelines to this region.

## Materials and methods

### Study type and design

COMIT-ANZ is a multicentre non-interventional retrospective study of patients undergoing LT for known or incidentally detected CCA or mixed HCC-CCA in Australia and New Zealand. The study included patients from the inception of LT programmes in ANZ in 1986 to December 2023. All included patients were aged 18 years and over.

Patients were identified using the ANZ Liver and Intestinal Transplant Registry (ANZLITR). Local transplant unit leads obtained retrospective clinical data pertaining to the transplant recipient and entered de-identified data into a REDCap database.

Data collection included (Supplementary Table 1): Patient demographics; transplant details; explant histology and related histological features of cancers; patient and graft survival; recurrence status and cause of death where relevant. The primary outcome of the study was 5-year overall survival and secondary outcomes included recurrence free survival.

### Recruitment procedure

Eligible recipients were identified using the ANZLITR. The de-identified ANZLITR registry number was provided to local site leads to facilitate re-identification of the patient. Local site leads collected data for included patients and in some cases also identified additional patients from their local database.

### Inclusion and exclusion criteria

Any adult patient undergoing LT for known or incidentally detected CCA or mixed HCC-CCA in one or more tumour nodules were included. Patients with other types of cancer such as colorectal liver metastasis were excluded.

### Ethics

Human research ethics approval (HREA) was sought and obtained from Austin Health with National Mutual Acceptance. This ethics applied to all included sites with the exception of Auckland City Hospital, New Zealand. Separate Australian based SSIs requested governance for their individual sites. The participating New Zealand site sought and obtained separate HREA approval through their local ethics office. In accordance with our ethics approval no consent was required as patients were identified retrospectively using the ANZLITR and local databases. This study was conducted according to the NHMRC National Statement on Ethical Conduct in Human Research (2007 and updates) as well as the World Medical Association Declaration of Helsinki (2013 and updates).

### Statistical methods

Data collected from this study was used to generate patient and graft survival Kaplan–Meier curves. Survival curves were compared using the log rank test. Cox regression analysis was performed used to identify factors associated with worse prognosis.

## Results

In total, 64 patients were identified as undergoing LT for either known or incidentally detected CCA or mixed HCC-CCA from within the ANZ region (Supplementary Table 2). Demographic information and past medical history for these patients is summarised in Table 1.

**Table 1:** Recipient demographics and past medical history.

	*n* (%)/95% IQR	Data available for *n* (%)
Gender		64 (100%)
Male	48 (75%)	
Female	16 (25%)	
Median Age at transplant (yrs)	55 (50–61)	64 (100%
Median Height (cms)	173 (165–178)	55 (86%)
Median Weight (kgs)	76 (61–91)	61 (95%)
Median MELD	14 (10–19)	50 (78%)
Median MELD-Na	15 (10–20)	33 (52%)
Diabetes mellitus	10 (16%)	63 (98%)
Hypertension	11 (17%)	63 (98%)
Ischaemic heart disease	5 (8%)	63 (98%)
Indication	Cholangiocarcinoma 15 (23%)	64 (100%)
	Hepatocellular carcinoma 13 (20%)	
	Primary sclerosing cholangitis 12 (19%)	
	Viral hepatitis 8 (13%)	
	Other 16 (25%)	

The majority of CCAs or mixed type HCC-CCAs were detected incidentally (75%) (Table 2). The majority of included patients had CCA (73%) (Table 2).

**Table 2:** Summary of tumour characteristics.

	*n* (%)
Timing of diagnosis	
Known tumour pre-transplant	16 (25%)
Incidentally detected tumour on explant histology^a^	48 (75%)
Unknown	0 (0%)
Site	
iCCA	27 (42%)
pCCA	21 (33%)
Unknown	16 (25%)
Histological Type	
CCA^b^	48 (75%)
HCC-CCA^b^	17 (27%)
Unknown	0 (0%)
Histopathological Lymphovascular Invasion	
Yes	29 (45.5%)
No	31 (48.5%)
Unknown	4 (6%)
Histopathological Perineural Invasion	
Yes	22 (34%)
No	30 (47%)
Unknown	12 (19%)

a Incidentally detected tumours were either mixed HCC-CCAs (*n* = 17) or iCCAs. No pCCAs were detected incidentally.

b One patient had nodules of both CCA and HCC-CCA.

**Figure 1:**
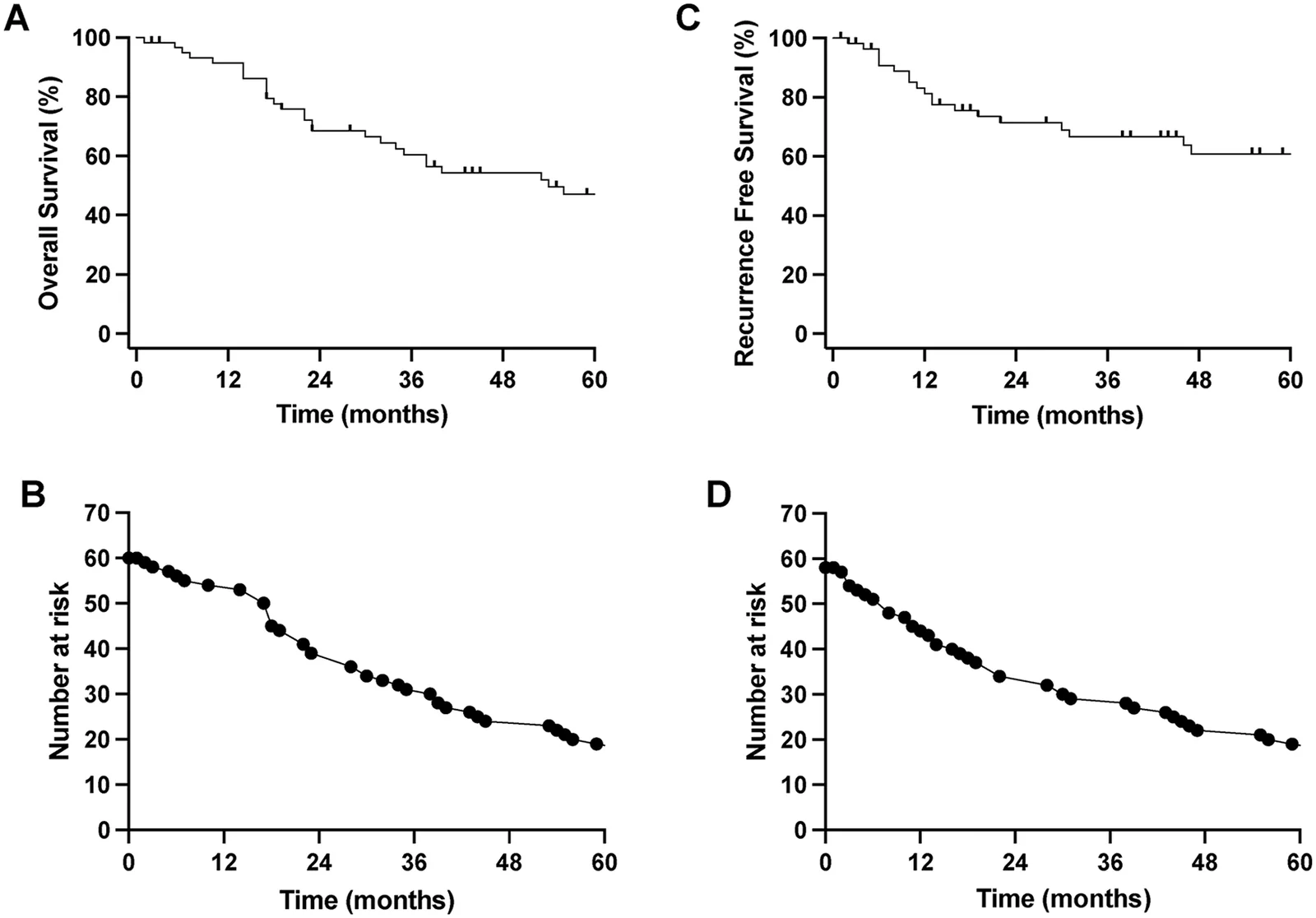
Survival data for All patients. **(A)** Overall Survival for all patients undergoing LT in ANZ for known CCA, incidentally detected CCA or mixed type HCC-CCA, *n* = 60. **(B)** Overall survival number at risk for all patients. **(C)** Recurrence Free Survival for all patients undergoing LT in ANZ for known or incidentally detected CCA or mixed type HCC-CCA, *n* = 59. **(D)** Recurrence free survival number at risk for all patients.

The median follow up time for all patients was 38 months (IQR: 18–237). The 5-year OS and 5-year RFS for all patients undergoing LT, for known CCA, incidentally detected CCA or mixed HCC-CCA, was 47% and 60% respectively (Figures 1A,C). The number at risk at 59 months was 19 patients for both OS and RFS (Figures 1B,D). By comparison, the 5-year OS for LT in ANZ is 83% and post LT for HCC is 79% in ANZ (ANZLITR).

### Survival according to timing of diagnosis

We examined survival according to whether the tumours were identified pre operatively and therefore “known” or incidentally detected incidentally on liver explant histology.

Survival data was captured for 16 patients undergoing LT for known CCA, the majority of these patients had underlying PSC (*n* = 11, 69%) and most of these tumours were perihilar CCAs (pCCA) (*n* = 10, 63%). Seven of the 10 patients with pCCA received neoadjuvant chemoradiotherapy. Radiotherapy was administered in the form of external beam radiotherapy and patients received 45–75 Gys. Chemotherapy was either single agent capecitabine or gemcitabine-cisplatin combined therapy. Of the 3 patients who did not receive neoadjuvant chemoradiotherapy, only one was historical to the era of neoadjuvant protocols for pCCA; the reasons for not giving the other two patients neoadjuvant therapy is unknown but these two patients were from the same centre. These two patients survived for 6 months and 35months, both experienced recurrence of disease and both died due to metastatic cholangiocarcinoma.

Median follow up for patients with known CCA was 25 months (IQR: 15–54 months). 1 year OS and RFS survival was 81% and 56% respectively. No patients achieved 5 year survival post LT for known CCA. Internationally, 5 year OS post LT for pCCA is reported at 53%–71% post neoadjuvant therapy (13, 23, 24).

**Figure 2:**
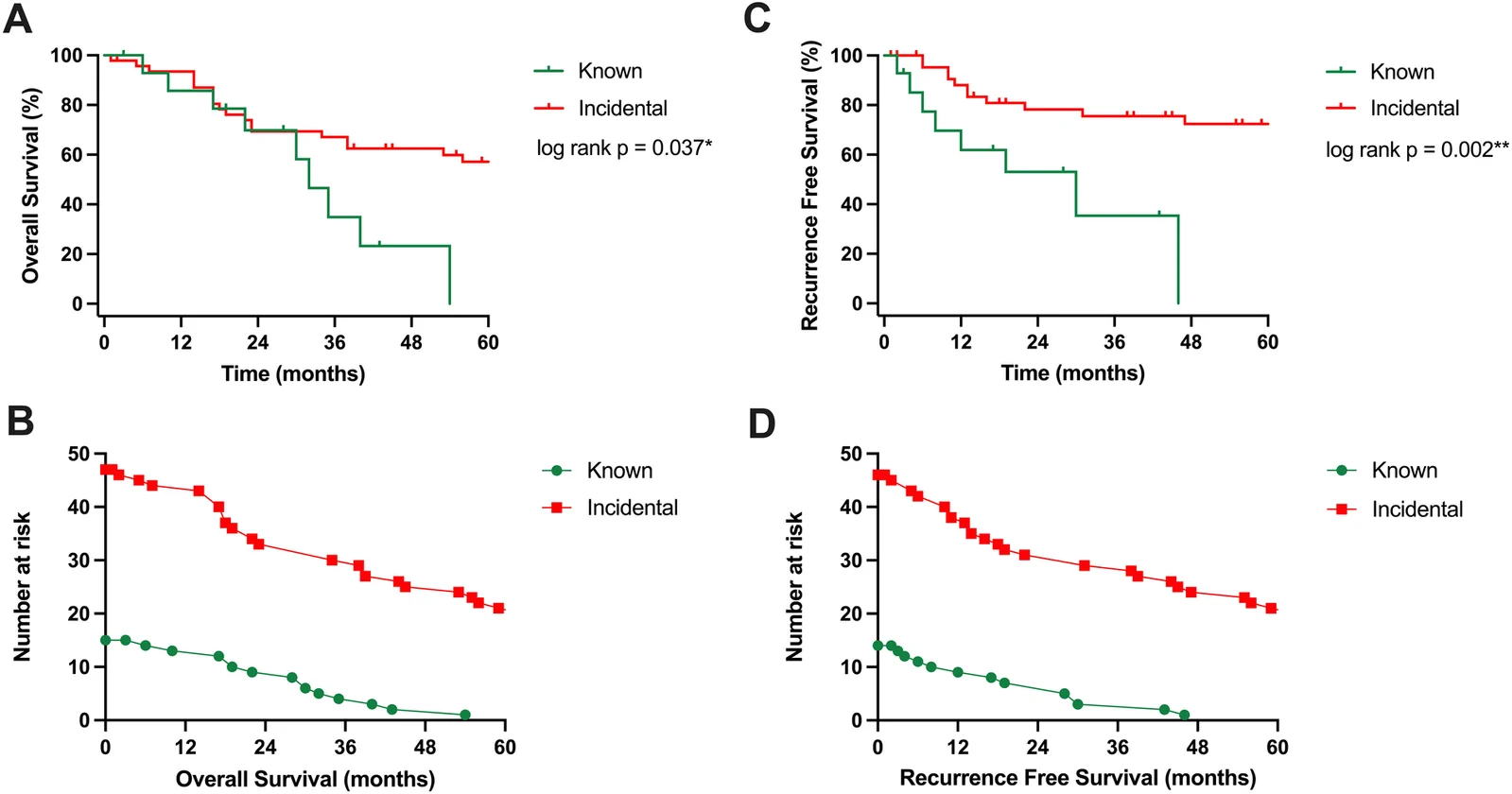
Survival for all patients according to time of diagnosis (known vs incidental). **(A)** Overall survival for patients with known (green) or incidentally detected tumours (red). **(B)** Overall survival number at risk for patients with known (green) or incidentally detected tumours (red). **(C)** Recurrence free survival for patients with known (green) or incidentally detected tumours (red). **(D)** Recurrence free survival number at risk for patients with known (green) or incidentally detected tumours (red).

The incidentally detected tumours included all the mixed HCC-CCAs (*n* = 17) and some iCCAs (*n* = 31), one patient had tumour nodules of both HCC and a mixed type HCC-CCA. No pCCAs were detected incidentally. 5-year OS and RFS for incidentally detected tumours (both CCAs and HCC-CCAs) was 57% and 68% respectively (Figures 2A–D) and was significantly improved compared to OS and RFS in the known group, *p* = .037 and *p* = .002 respectively (Figures 2A–D).

We also repeated this analysis but included only the CCAs, (all incidentally detected mixed type HCC-CCAs we removed from this analysis) and the results remained very similar (Supplementary Figure 1).

### Survival according to histological subtype

**Figure 3:**
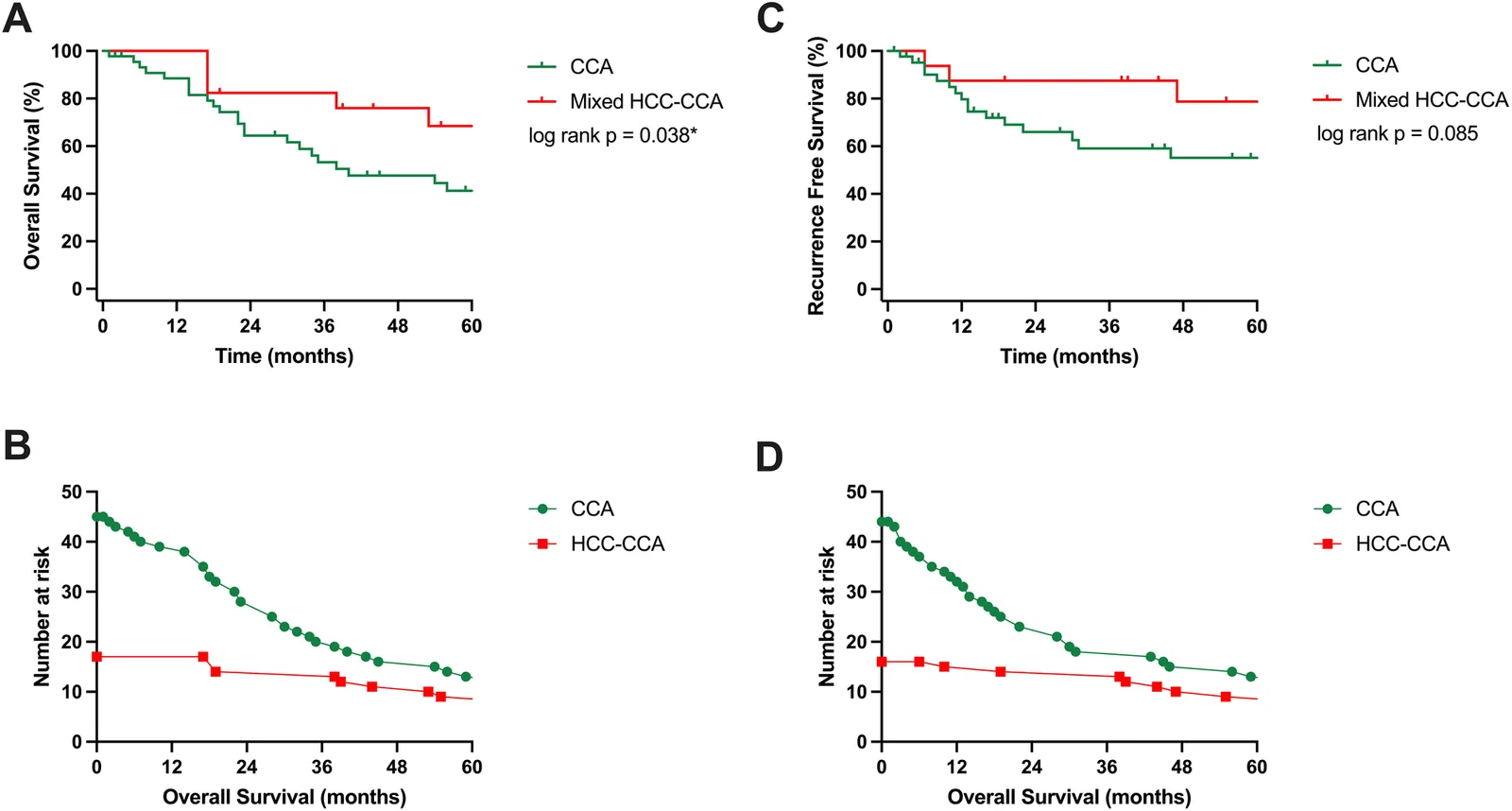
Survival according to histological subtype (CCA vs mixed type HCC-CCA). **(A)** Overall survival for patients undergoing LT for cholangiocarcinoma (green) and mixed type HCC-CCAs (red). **(B)** Overall survival number at risk for patients undergoing LT for cholangiocarcinoma (green) and mixed type HCC-CCAs (red). **(C)** Recurrence free survival for patients undergoing LT for cholangiocarcinoma (green) and mixed type HCC-CCAs (red). **(D)** Recurrence free survival number at risk for patients undergoing LT for cholangiocarcinoma (green) and mixed type HCC-CCAs (red).

We were also interested to investigate how the patients with mixed type HCC-CCAs fared post liver transplant, compared to the CCAs as there is a paucity of data regarding survival post liver transplant for mixed HCC-CCAs. All of the mixed HCC-CCAs were incidentally detected on liver explant histology (*n* = 17). 5-year OS for CCAs was 51%, vs. 68% for the mixed type HCC-CCAs (*p* = .038*) (Figures 3A–D). 5-year RFS for the CCAs was 55%, vs. 78% for the mixed type HCC-CCAs (*p* = .085) (Figure 3C).

### Survival according to site

**Figure 4:**
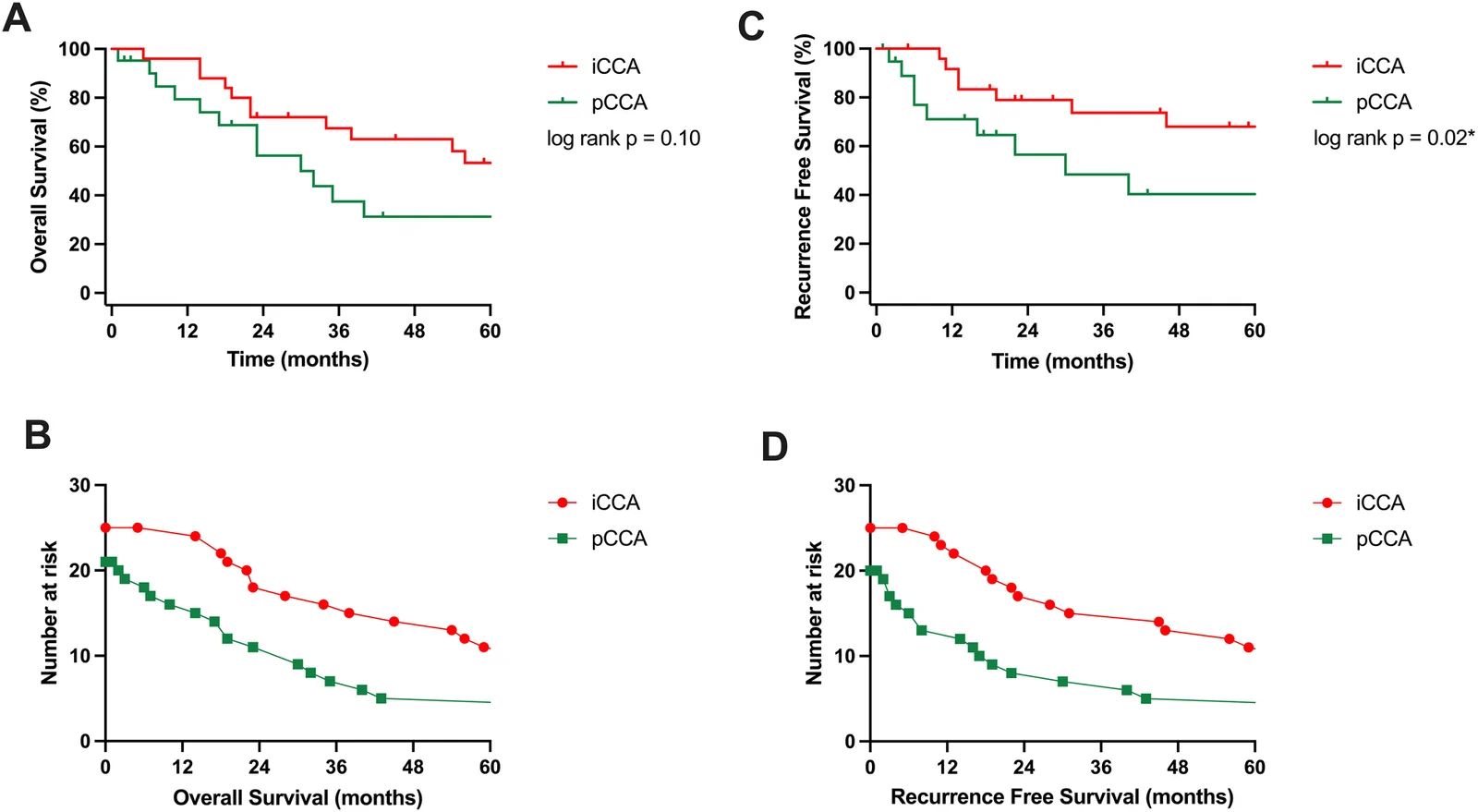
Survival according site (pCCA vs iCCA). **(A)** Overall survival for patients undergoing LT for iCCA (red) and pCCA (green). **(B)** Overall survival number at risk for patients undergoing LT for iCCA (red) and pCCA (green). **(C)** Recurrence free survival for patients undergoing LT for iCCA (red) and pCCA (green). **(D)** Recurrence free survival number at risk for patients undergoing LT for iCCA (red) and pCCA (green).

We also investigated whether patient survival differed between the iCCAs (*n* = 27) and the pCCAs (*n* = 21). 5-year OS for patients with iCCA was 53%, compared to 31% for patients with pCCA (Figure 4A). RFS was significantly worse for patients undergoing LT for pCCA compared to iCCA (*p* = .020) (Figure 4B). 5-year RFS was 68% for patients with iCCA, compared to 40% for pCCA (Figure 4C). There were 7 recurrences amongst the iCCA group and the majority occurred within the first 20 months post-transplant. There were 10 recurrences amongst the pCCA group and these occurred at a wide range of time points, ranging from 2 to 134 months post-transplant.

### Survival according to size

**Figure 5:**
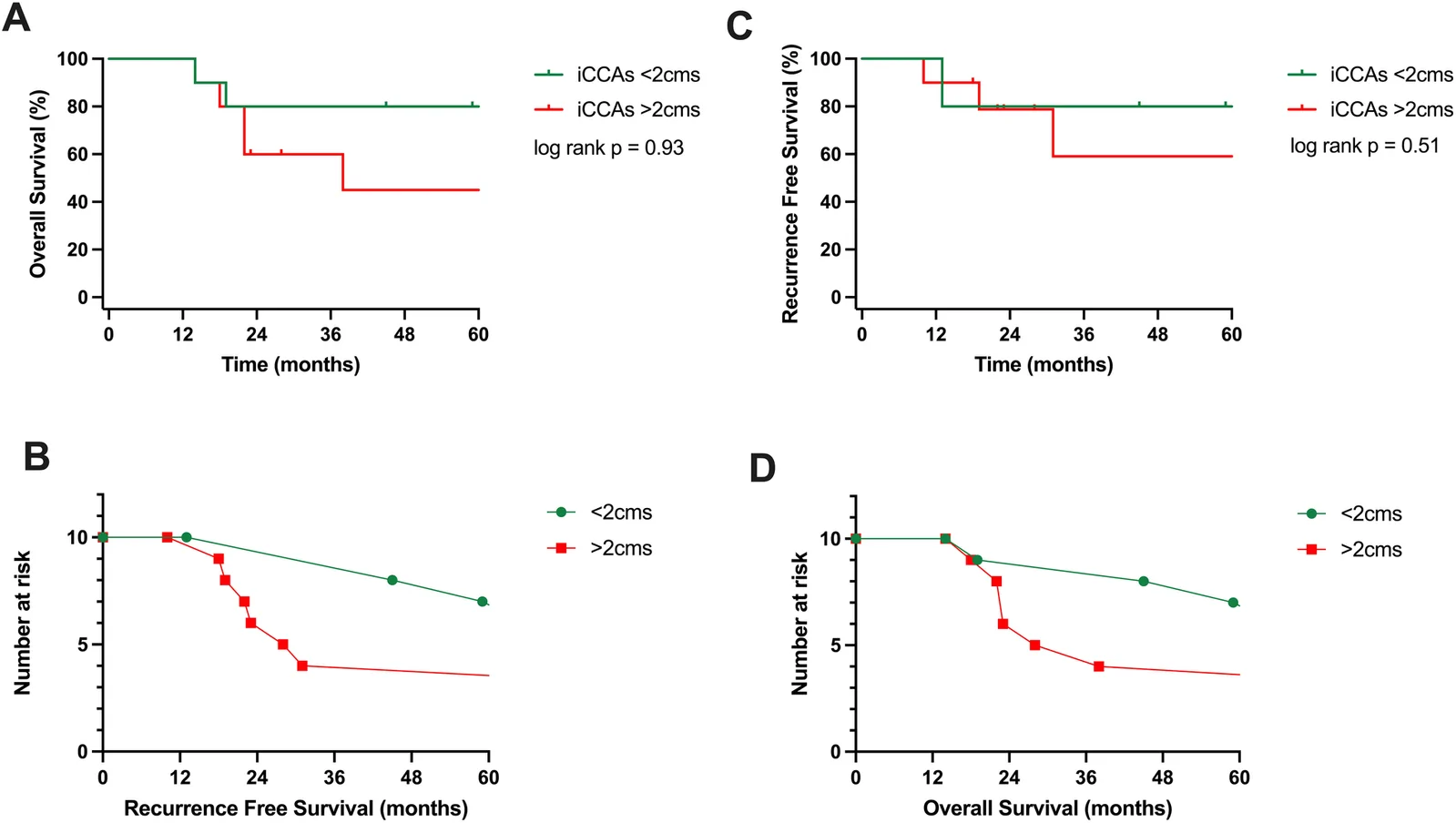
Survival according to iCCA size. **(A)** Overall survival for patients undergoing LT for iCCAs >2 cms (red) and iCCAs <2 cms (green). **(B)** Overall survival number at risk for patients undergoing LT for iCCAs >2 cms (red) and iCCAs <2 cms (green). **(C)** Recurrence free survival for patients undergoing LT for iCCAs >2 cms (red) and iCCAs <2 cms (green). **(D)** Recurrence free survival number at risk for patients undergoing LT for iCCAs >2 cms (red) and iCCAs <2 cms (green).

In order to more directly assess the applicability of the most recent ILTS guidance, we also analysed survival according to cholangiocarcinoma size. Whilst there were only small numbers to analyse, iCCAs <2 cms (*n* = 10) and iCCAs >2 cms (*n* = 11) there is a strong trend to much better survival in the <2 cms group, in accordance with the current international guidance (Figures 5A–D).

### Univariate and multivariate analysis

In order to identify patient factors associated with poorer overall survival, a univariate Cox analysis was performed including all patients included in this study (Table 3). Multiple variables were examined according to overall survival (Table 3). This demonstrated that age (*p* < 0.01), known tumours (*p* = 0.022), mixed type HCC-CCAs (*p* = 0.040) and poorly differentiated tumours (*p* = .026) were associated with poorer outcomes (Table 3).

**Table 3:** Univariate Cox analysis of patient factors which may or may not be associated with poorer overall survival.

Variable	HR (95% CI)	*p* value
Age	0.948 (0.912–0.987)	**0** **.** 008**
Sex	0.726 (0.360–1.585)	0.392
Diabetes mellitus	0.906 (0.337–2.050)	0.827
Hypertension	1.088 (0.404–2.489)	0.852
Ischaemic heart disease	0.850 (0.203–2.393)	0.788
MELD	0.995 (0.940–1.047)	0.860
Known vs Incidental	2.530 (1.1.05–5.497)	**0** **.** 022*
Mixed type vs CCA	0.340 (0.149–0.894)	**0** **.** 040*
LVI	0.574 (0.285–1.154)	0.117
PNI	2.103 (0.927–4.754)	0.071
Poorly differentiated	3.105 (1.158–8.821)	**0** **.** 026*
Maximal diameter	1.005 (0.975–1.033)	0.736
Tumour Size >2 cms	1.159 (0.507–2.582)	0.720
PSC	1.183 (0.597–2.297)	0.622
Rejection post transplant	0.715 (0.353–1.410)	0.338
mTOR post transplant	1.045 (0.458–2.180)	0.910

Significant values shown in bold. *p* values <0.20 were included in the multivariate analysis.

From the univariate analysis, variables with *p* < 0.20 were used for a multivariate analysis. Multivariate Cox analysis found that the only factor which remained significantly associated with poorer overall survival was poorly differentiated tumours (*p* = 0.021) (Table 4). Survival curve analysis, comparing overall survival for patients with well, moderately and poorly differentiated tumours did not differ significantly but did highlight that well to poorly differentiated tumours are associated with consequential reductions in overall survival (Supp Figure 4).

**Table 4:** Multivariate Cox analysis.

Variable	HR (95% CI)	*p* value
Age	1.003 (0.942–1.080)	0.931
Known vs Incidental	2.159 (0.385–15.36)	0.416
Mixed type vs CCA	0.128 (0.007–0.727)	0.058
LVI	1.250 (0.466–3.363)	0.654
PNI	0.811 (0.224–2.556)	0.733
Poorly differentiated	4.783 (1.291–18.96)	**0** **.** 021*

Multivariate Cox analysis was performed using variables which has *p* < 0.20 on the univariate analysis. Significant variables are shown in bold.

The all comer, 5-year OS was 47%. For patients undergoing LT for iCCAs <2 cms, the 5-year OS and 5-year RFS were both 80%. Poorly differentiated tumours were associated with significantly poorer overall survival (Supplementary Figure 2).

## Discussion

This nationwide study of clinical outcomes following LT for known or incidentally detected CCA and mixed HCC-CCAs in Australia and New Zealand supports LT for very small intrahepatic CCAs <2 cms which can achieve excellent long term survival, in keeping with current international guidance (17, 25).

This study demonstrated that within the ANZ region, 5-year OS for patients undergoing LT for cirrhosis with very small iCCAs (<2 cms) was 80%. Whilst there is no level 1 data to support LT in the setting of iCCA, multiple studies have shown that small (<2 cms) or early stage (T1) intrahepatic cholangiocarcinomas achieve excellent long term survival following liver transplant (15, 26). Practically this advice is quite important, as it is not infrequent that patients with cirrhosis, either on a background of PSC or not, develop intrahepatic masses that can be difficult to diagnose during their transplant work up. Differentiating regenerative nodules, HCCs and small iCCAs is notoriously tricky and importantly this data supports LT in the setting of indistinguishable small masses that could on explant histology be proven to be iCCAs.

In addition to iCCA size, other factors were also associated with poorer outcomes. The data presented in this study demonstrates that poorly differentiated tumours were also significantly associated with worse clinical outcomes. In future, this could inform decision making regarding appropriateness for liver transplantation whereby if a pre-operative biopsy is able to provide a diagnosis of poorly differentiated CCA then patients could be managed along a different treatment path. However, the role of pre-operative biopsies in risk stratifying CCAs requires further interrogation with future studies.

There is a scarcity of data regarding LT for patients with mixed HCC-CCAs. These tumours are also very difficult to accurately diagno pre-transplantation due to reasons already discussed in this paper—lack of pathognomic radiological features and sampling error. This is reflected in the data presented here; all included mixed HCC-CCAs were detected incidentally on liver explant histology and none were knowingly transplanted. Furthermore, mixed HCC-CCAs are not an indication for LT currently. A multi-centre American study of 67 patients who underwent LT for mixed HCC-CCAs within Milan criteria found that compared to HCC, transplant recipients had similar 5-year OS (HCC-CCA 70.1% vs. HCC 73.4%) and DFS (27). In this study, we demonstrated a comparable 5-year OS of 68% for patients undergoing LT for mixed HCC-CCAs.

There are some significant limitations of this study which need to be addressed. This study is retrospective and due to the long follow up time, there have been significant advances and changes to clinical practise. The retrospective nature of the study meant that not all the data points were available for all patients and this had ramifications on the completeness of the statistical analysis. Additionally, significant heterogeneity exists; some recipients were transplanted knowingly for perihilar CCA or intrahepatic CCA, others were detected incidentally on explant histology. We also chose to include mixed HCC-CCAs into this study. Despite these limitations this study does highlight that the current international guidance pertaining to the management of very early iCCAs does apply to our region and that patients undergoing LT for cirrhosis with very early iCCAs have excellent 5-year overall survival.

## Data Availability

The raw data supporting the conclusions of this article will be made available by the authors, without undue reservation.

## Prometheus collaborative members list

Professor Vijayaragavan Muralidharan; Professor Robert Jones; Professor Marcos V Perini; Professor Mehrdad Nikfarjam; Associate Professor Michael Fink; Dr Graham Starkey; Dr Bao Zhong Wang; Dr Eunice Lee; Dr Ruelan Furtado; Dr Osamo Yoshino; Dr Su Kah Goh.
