# Supplementary material for: COMIT-ANZ: an audit of cholangiocarcinoma and mixed hepatocellular carcinoma-cholangiocarcinoma outcomes for patients undergoing liver transplantation in Australia and New Zealand
Source: Front Transplant. 2026 Aug 7;5:1856922. doi: 10.3389/frtra.2026.1856922 (PMC13493370; doi:10.3389/frtra.2026.1856922)
Supplement: Supplementary file 1 [file Table1.docx]

**Supplementary Materials**

Supp Figure 1. Kaplan Meier survival curves for known and incidentally detected CCAs only (mixed type HCC-CCAs excluded from this analysis).

Supp Figure 2. Overall survival according to tumour differentiation for all comers.

Data to be collected

DEMOGRAPHICS

Gender

Age at transplant

MELD

MELD-Na

Diabetes mellitus

IHD

HTN

BMI

PRE-TRANSPLANT DISEASE

Pre-Transplant Radiology

Number of tumour nodules

Maximal size of tumour nodule

AFP at transplant

CEA at transplant

Ca19.9 at transplant

Pre-Transplant Histology

Biopsy histology

PRE-TRANSPLANT TREATMENT

For known CCA

Neo-adjuvant therapy (NAT)

Type of NAT

Duration of NAT

Date ceased NAT

For HCC

TACE

RFA

MWA

PEI

Any other loco-regional therapy?

TRANSPLANT VARIABLES

Date of LT

Transplant indication

Incidental CCA

Incidental mixed CCA/HCC

Explant Histology

Number of HCC nodules

Number of CCA nodules

Number of mixed HCC/CCA nodules

Maximal diameter of CCA/mixed HCC-CCA

Differentiation (well, moderate, poor)

Classification of CCA (intrahepatic, perihilar)

Perineural invasion

Lymphovascular invasion

T staging (AJCC 2017 8^th^ edition) for hilar/perihilar

POST-TRANSPLANT TREATMENT

Immunosuppression regime at induction

mTOR Inhibitor (Y/N)

Duration of mTOR inhibitor

Transplant rejection (Y/N)

Pulsed steroids (Y/N)

Increase or change in immunosuppression

TRANSPLANT OUTCOMES

Date of death

Cause of death

Graft survival date (date or re-tx or date of last follow up)

Graft failure reason

Re-transplant indication

Tumour recurrence (Y/N)

Date of tumour recurrence (date recurrence dx radiologically/histologically)

Local or metastatic recurrence

Recurrence treatment

Treatment intent

**Supp Table 1. Data to be collected**

|  | **n = 64** |
| --- | --- |
| **Queensland** | **20** |
| **New South Wales** | **17** |
| **Victoria** | **16** |
| **New Zealand** | **6** |
| **South Australia** | **3** |
| **Western Australia** | **2** |

**Supp Table 2. Patients included from different states and regions.**
